# Building an Asymmetrical Brain: The Molecular Perspective

**DOI:** 10.3389/fpsyg.2019.00982

**Published:** 2019-04-30

**Authors:** Judith Schmitz, Onur Güntürkün, Sebastian Ocklenburg

**Affiliations:** Biopsychology, Department of Psychology, Institute of Cognitive Neuroscience, Ruhr University Bochum, Bochum, Germany

**Keywords:** laterality, handedness, language lateralization, epigenetics, Nodal pathway

## Abstract

The brain is one of the most prominent examples for structural and functional differences between the left and right half of the body. For handedness and language lateralization, the most widely investigated behavioral phenotypes, only a small fraction of phenotypic variance has been explained by molecular genetic studies. Due to environmental factors presumably also playing a role in their ontogenesis and based on first molecular evidence, it has been suggested that functional hemispheric asymmetries are partly under epigenetic control. This review article aims to elucidate the molecular factors underlying hemispheric asymmetries and their association with inner organ asymmetries. While we previously suggested that epigenetic mechanisms might partly account for the missing heritability of handedness, this article extends this idea by suggesting possible alternatives for transgenerational transmission of epigenetic states that do not require germ line epigenetic transmission. This is in line with a multifactorial model of hemispheric asymmetries, integrating genetic, environmental, and epigenetic influencing factors in their ontogenesis.

## Introduction

**Figure 1:**
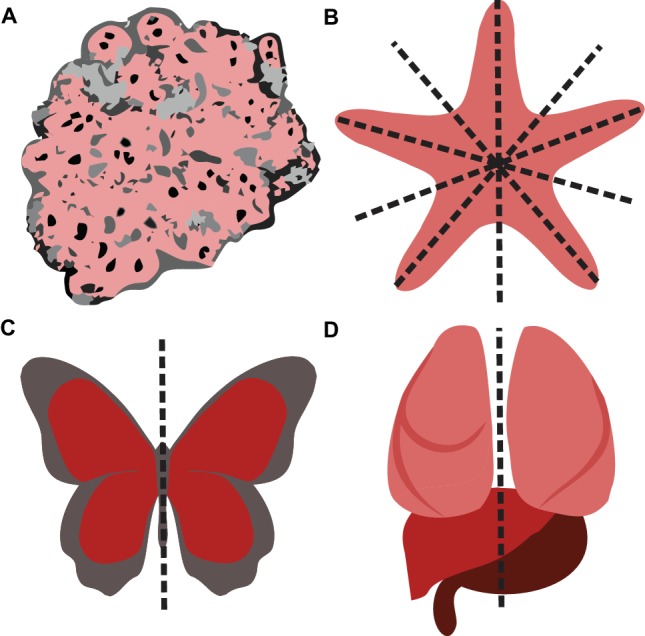
The major types of body plans. **(A)** Asymmetry. **(B)** Radial symmetry. **(C)** Bilateral symmetry. **(D)** Due to the asymmetrical position of internal organs, humans and other vertebrates have been described as pseudo-bilateral (Levin, 2005).

In 1866, German zoologist Ernst Haeckel introduced promorphology – the science of an organism’s external form – and proposed symmetry as a fundamental criterion for classifying organisms (Haeckel, 1866). The clade of bilateria (animals displaying mirror-inverted body halves), including (but not restricted to) all vertebrates, was created in 1888 (Hatschek, 1888). Besides asymmetry (organisms without any axis or plane of symmetry, e.g., the majority of sponges) and radial symmetry (organisms with one axis, but several planes of symmetry, e.g., starfish), bilateral symmetry is considered one of the three major types of body plans (Manuel, 2009). However, bilateral symmetry is frequently broken by either the position of non-paired internal organs in one body half (e.g., the left-sided stomach and the right-sided liver) or by anatomical differences between the left and right half of paired internal organs. For example, the human lungs are constituted of two lobes on the left and three lobes on the right side. Based on these observations, humans and other vertebrates have also been described as “pseudo-bilateral” (see Figure 1; Levin, 2005).

The brain is one of the most striking examples for structural and functional differences between the left and right half of the body. Functional hemispheric asymmetries are found in several aspects of cognition such as memory, emotion, attention, language, and executive functions (Ocklenburg et al., 2014b). This review article is aimed at elucidating the molecular factors underlying the asymmetrical development of the human brain. We recently reviewed the evidence for environmental factors in handedness ontogenesis and suggested that functional hemispheric asymmetries such as handedness are partly under epigenetic control (Schmitz et al., 2017b). However, in a fast-moving field like genetics, significant progress has been made in the meantime. First empirical studies reported the effects of DNA methylation on the strength of functional hemispheric asymmetries in the individual (Leach et al., 2014; Schmitz et al., 2018a,b). Here, we aim to focus on the relationship of functional hemispheric asymmetries with inner organ asymmetries. Moreover, we previously (Schmitz et al., 2017b) suggested that epigenetic mechanisms might partly account for the large gap between heritability estimates for handedness from twin and adoption studies of up to 0.66 (Risch and Pringle, 1985) and the small variance explained by molecular genetic studies (Eriksson et al., 2010; Armour et al., 2014), also known as the missing heritability problem (Maher, 2008). However, in order to account for this gap, this mechanism requires transgenerational transmission of epigenetic states in families. As germ line epigenetic inheritance is highly controversial in humans (Ambeskovic et al., 2017b), we suggest an alternative mechanism by which epigenetic mechanisms might account for the missing heritability of functional hemispheric asymmetries.

## The Development of Asymmetry

The consistency of population-level lateralization in handedness across centuries (Faurie and Raymond, 2004) and continents (Raymond and Pontier, 2004) is likely the result of asymmetrical prenatal CNS development (Willems et al., 2014). Recent evidence suggests a molecular genetic association of handedness with the ontogenesis of visceral asymmetries (Brandler et al., 2013; Brandler and Paracchini, 2014). As handedness and language lateralization are not completely independent from each other (Knecht et al., 2000; Jansen et al., 2007; Somers et al., 2015a), the same could hold true for language lateralization. Thus, to investigate the molecular factors underlying the ontogenesis of functional hemispheric asymmetries and a possible relationship with visceral asymmetries, it is important to consider the emergence of visceral as well as structural and functional hemispheric asymmetries in human development.

### The Emergence of Visceral Asymmetries

**Figure 2:**
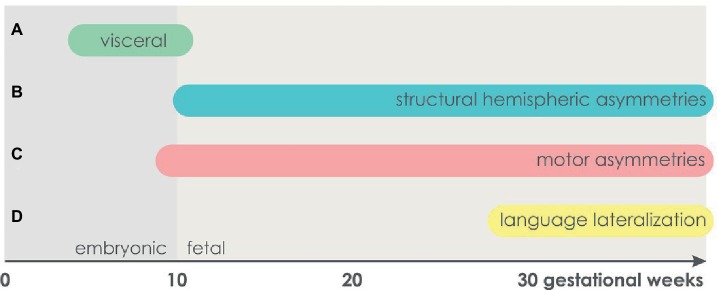
The time course of asymmetry development. **(A)** Visceral asymmetries. **(B)** Structural hemispheric asymmetries. **(C)** Motor asymmetries. **(D)** Language lateralization.

The human body is symmetric at the beginning of embryonic development. The first visceral asymmetry is detected when the heart, initially a straight tube, starts to loop at the end of 5 gestational weeks and occupies its typical left-sided position (Steding, 2009). During an embryonic twist along the rostrocaudal axis, asymmetries of other organs emerge (Kathiriya and Srivastava, 2000). The liver is larger on the right than the left side at 6 gestational weeks (Hutchins and Moore, 1988). The lung divides into the left and right lung buds differing in length at 7 gestational weeks. One week later, the lung buds develop three lobes in the right and two lobes in the left body half (Steding, 2009). The development of the stomach is characterized by left-convex bending starting at 7 gestational weeks. While the liver extends massively, the stomach grows into its characteristic left-sided position (Steding, 2009). Overall, the establishment of visceral asymmetries is completed at the end of the embryonic period [end of 10 gestational weeks (see Figure 2A; O’Rahilly, 1979)].

### The Emergence of Structural Hemispheric Asymmetries

Like the visceral organs, the brain develops from an unpaired structure, the neural tube. Initially, it mostly grows ventrally, resulting in the emergence of its main subregions prosencephalon, mesencephalon, and rhombencephalon at the age of 6 gestational weeks. The cerebral hemispheres differentiate toward the end of 8 gestational weeks (Chi et al., 1977a; Steding, 2009). The earliest structural hemispheric asymmetry that has been found to date is an enlargement of the left compared to the right choroid plexus in terms of length, area, and circumference at 11–13 gestational weeks (Abu-Rustum et al., 2013). Thus, in contrast to visceral asymmetries which are established in the embryonic period, structural hemispheric asymmetries do not seem to emerge before the fetal period starting at the 11th gestational week (see Figure 2B; O’Rahilly, 1979). As suggested by Corballis (2013), the early structural asymmetry in the choroid plexus might be a precursor for other hemispheric asymmetries. Specifically, interhemispheric differences in the production of cerebrospinal fluid, which synthesizes peptides, growth factors, and cytokines, might underlie the pronounced leftward planum temporale asymmetry evident at the 31st gestational week (Chi et al., 1977b; Kasprian et al., 2011). Starting at the 20th gestational week, a leftward asymmetry in cortical volume has been reported for the occipital lobes (Weinberger et al., 1982) as well as for the entire left hemisphere (Hering-Hanit et al., 2001; Kivilevitch et al., 2010; Andescavage et al., 2017).

### The Emergence of Motor Asymmetries

In line with structural hemispheric asymmetries already being apparent in human fetal development, prenatal ultrasound studies indicated early signs of motor asymmetries in terms of head turning and arm movements. The majority of human fetuses display a strong preference for turning the head toward the right side. Ultrasound observations of fetuses positioned in the cephalic (head-first) presentation revealed a right side preference between 30 and 38 gestational weeks (Ververs et al., 1994). While this pattern was confirmed in a subsequent study, a midline preference was observed in breech-positioned (feet-first) fetuses (Fong et al., 2005). Newborns also show a population-level asymmetry toward turning the head to the right side (Dunsirn et al., 2016), and the preferred direction of head turning predicts hand use in reaching tasks in infants (Coryell and Michel, 1978; Michel, 1981; Konishi et al., 1986). Congenital muscular torticollis involves unilaterally shortened neck muscles, which leads to a permanent head tilt sustaining visual experience toward either the left or right hand. Ocklenburg et al. (2010) found a strong impact of sustained visual experience toward one hand on the probability of being right- or left-handed in children affected by this disorder. In adults, 64.5% of kissing couples turn their head toward the right side, indicating a persistence of head turning bias into adulthood (Güntürkün, 2003). Moreover, the preferred side of head turning is correlated with handedness LQ (Ocklenburg and Güntürkün, 2009). Overall, the tendency to turn the head toward one side is likely to be associated with motor preferences in later life.

Using ultrasound, individual arm movements are detectable at 9 gestational weeks (de Vries et al., 2001). Starting at the 10th gestational week, a strong preference of right arm movements is apparent in three quarters of fetuses (Hepper et al., 1998), which is persistent throughout fetal development (see Figure 2C; McCartney and Hepper, 1999). Starting at the 15th gestational week, 90% of fetuses prefer right-sided thumb sucking. This early preference is highly persistent as it is correlated with head turning preference after birth (Hepper et al., 1990, 1991) and handedness at school age (Hepper et al., 2005). The finding of a prenatal hand preference that is consistent until school age was lately confirmed by kinematic analysis of fetal arm movements: In fetal development, individuals acted faster and more precisely using the hand that they would report as their dominant hand at age 9. By analyzing movement or deceleration time for touching the eye or mouth, subsequent handedness could be classified with an accuracy between 89 and 100% (Parma et al., 2017).

### The Emergence of Language Lateralization

Language lateralization in newborns and infants has mainly been studied in terms of perceptional asymmetries. At the behavioral level, a right ear advantage for dichotically presented syllables has been found in infants (Entus, 1980) and 4-day-old neonates (Bertoncini et al., 1989). Moreover, greater left-hemispheric temporal activation in response to language stimuli has been reported in neuroimaging studies within the first postnatal week (Peña et al., 2003; Gervain et al., 2008). In order to investigate language lateralization as early as possible, preterm infants between 28 and 30 gestational weeks were tested with a linguistic (typically left hemisphere dominant) and a non-linguistic (typically right hemisphere dominant) discrimination task. For linguistic discrimination, functional optical imaging revealed that posterior temporal areas showed faster and more sustained activation in the left hemisphere. The left frontal region (Broca’s area) was responsive to linguistic, but not to non-linguistic discrimination (see Figure 2D; Mahmoudzadeh et al., 2013). In contrast, pitch processing has been shown to be right-lateralized in fetuses and preterm infants (Schleussner et al., 2004; Mento et al., 2010). However, Perani et al. (2011) found rather bilateral processing of language perception in neonates. For word production as assessed by fTCD, it has been shown that only 60% of 6- to 11-year-old children, but 95% of adults, showed left-hemispheric language lateralization (Haag et al., 2010). Thus, it has been assumed that language lateralization develops in the course of language acquisition (Minagawa-Kawai et al., 2011; Bishop, 2013).

## The Role of Genetics and Gene Expression

The fact that functional hemispheric asymmetries are already established in the human fetus is in line with a genetic influence (Hepper, 2013). The observation that handedness direction is more similar within than between families has inspired genetic theories of handedness since the early twentieth century (Ramaley, 1913). Early genetic models assumed one gene with two alleles to establish handedness and language lateralization (Annett, 1964; McManus, 1985). However, after sequencing of the human genome, molecular genetic studies on functional hemispheric asymmetries revealed a far more complex picture of the underlying molecular factors.

### The Genetics of Hemispheric Asymmetries

The neurogenetics of handedness has been reviewed in detail elsewhere (Ocklenburg et al., 2013c). Shortly, two genome-wide association studies (GWAS) did not reveal any single nucleotide polymorphism (SNP) associated with handedness direction (Eriksson et al., 2010; Armour et al., 2014). A linkage study in a Dutch population isolate confirmed this result but found suggestive linkage for handedness in in chromosomal region 22q13 (Somers et al., 2015b). Candidate genes for handedness include the androgen receptor gene (*AR*) (Medland et al., 2005; Hampson and Sankar, 2012; Arning et al., 2015), the catechol-O-methyltransferase gene (*COMT*) (Savitz et al., 2007), the leucine-rich repeat transmembrane neuronal 1 gene (*LRRTM1*) (Francks et al., 2007), and the *PCSK6* gene (proprotein convertase subtilisin/kexin type 6) (Scerri et al., 2011; Arning et al., 2013; Brandler et al., 2013). Two recent studies found an association of handedness with the *SETDB2* gene (SET domain, bifurcated 2) encoding for a methyltransferase regulating hemispheric asymmetries in the zebrafish model (Ocklenburg et al., 2016a; Crespi et al., 2018). Overall, handedness is likely to be a complex, polygenic trait. However, several large-scale twin studies estimated 24–26% of phenotypic variance to be explained by genetic factors with the remainder being influenced by shared and unique environment (Medland et al., 2006, 2009; Vuoksimaa et al., 2009). Thus, as pointed out by Hepper (2013), the early emergence of handedness in fetal development is in line with a genetic effect on the initial appearance but does not exclude an effect of perinatal and postnatal environmental factors on handedness.

To date, no GWAS for language lateralization has been performed yet. Early twin studies found no correlation between dichotic listening task performances of monozygotic twins and concluded an absence of genetic effects on language lateralization (Springer and Searleman, 1978; Jäncke and Steinmetz, 1994). In twin pairs concordant for handedness, the correlation of language lateralization quotients was 0.74, suggesting a genetic component. However, in twin pairs discordant for handedness, the correlation was only 0.18 (Sommer et al., 2002). Bryden (1975) reported a positive correlation between maternal and offspring lateralization, but no such correlation between paternal and offspring lateralization. In a more recent study, Ocklenburg et al. (2016c) found no heritability for language lateralization determined by the dichotic listening task. In contrast, there was significant heritability for attentional modulation of language lateralization (Ocklenburg et al., 2016c). In contrast, a genetic linkage study estimated the heritability for language lateralization based on fTCD to be 0.31 (Somers et al., 2015b). Based on an elevated incidence of atypical language lateralization in schizophrenia, Crow (2008) proposed shared genetic mechanisms between schizophrenia and functional hemispheric asymmetries. The “Big Bang” theory suggests that a genetic speciation event involving Protocadherin11X and Y gave rise to the development of hemispheric asymmetries and human language, while an absence of hemispheric asymmetries is reflected in schizophrenic symptoms. However, a large-scale GWAS did not confirm a role of this gene pair in schizophrenia (Schizophrenia Working Group of the Psychiatric Genomics Consortium, 2014). Molecular genetic evidence regarding candidate gene for language lateralization suggest a role of the Forkhead box P2 gene (*FOXP2*) (Pinel et al., 2012; Ocklenburg et al., 2013b), the *KIAA0319/TTRAP/THEM2* locus (Pinel et al., 2012), the NMDA receptor 2B subunit gene (*GRIN2B*) (Ocklenburg et al., 2011) and the Cholecystokinin A receptor gene (*CCKAR*) (Ocklenburg et al., 2013a). The association of the Proteolipid Protein 1 gene (*PLP1*) with language lateralization suggests modulation *via* the *corpus callosum* (Ocklenburg et al., 2018). Overall, although to date no specific environmental factors have been associated with language lateralization, the fact that several studies in newborns and infants find rather bilateral processing of language (Bishop, 2013) and the moderate heritability estimates from twin and family studies suggest that environmental factors also contribute to the development of language lateralization.

### The Molecular Link Between Visceral and Hemispheric Asymmetries

**Figure 3:**
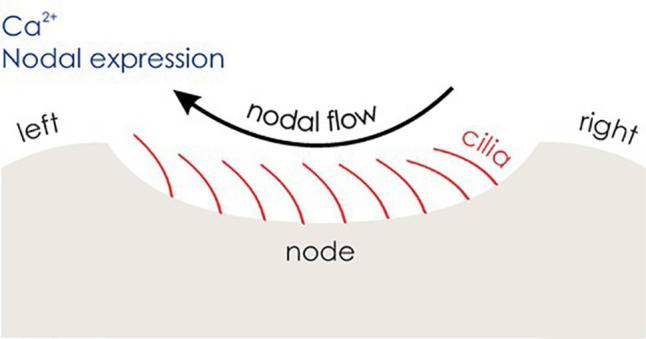
The development of visceral asymmetries. Motile cilia (red) induce a leftward nodal flow, which is transduced into stronger left-sided Ca^2+^ signaling and Nodal expression, triggering the Nodal signaling pathway in the left lateral plate mesoderm (LPM).

Over the past years, there has been evidence for a molecular genetic link of handedness with the development of body asymmetries (Brandler et al., 2013; Brandler and Paracchini, 2014; Schmitz et al., 2017a). Visceral asymmetries at the structural level are preceded by a cascade of molecular events leading to an asymmetric body plan in the embryo. A universal model of visceral asymmetry development in vertebrates has been established over the course of the recent years. An initial symmetry break starts around 8 days embryonic age in mice. At this early stage of development, the vertebrate embryo has an elongated body form with the head and heart at the anterior and a cavity known as the node at the posterior end. Within the node, the clockwise rotational movement of motile cilia (hair-like cell organelles with the ability to beat) induces a leftward flow of extracellular fluid (Nonaka et al., 1998). In a second step, Pkd2 (polycystic kidney disease 2) transduces this leftward nodal flow into stronger left-sided Ca^2+^ signaling on the edge of the node (McGrath et al., 2003; Takao et al., 2013) as well as stronger left-sided expression of Nodal (see Figure 3). Nodal is an intercellular signaling protein of the transforming growth factor beta (TGF-beta) family (Zhou et al., 1993).

**Figure 4:**
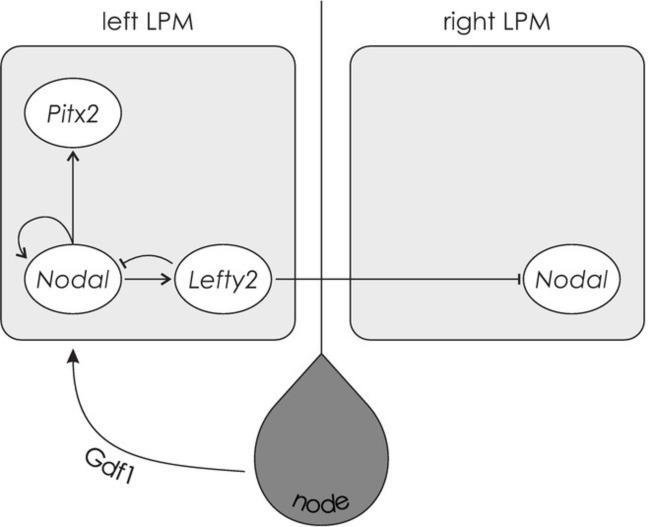
The Nodal signaling cascade. Facilitated by Gdf1, Nodal is transmitted to the left LPM, expressing *Nodal*, *Lefty2*, and *Pitx2*. The protein encoded by *Lefty2* suppresses the Nodal pathway in the right LPM. *Pitx2* encodes a transcription factor that is involved in asymmetric morphogenesis.

With the help of the growth/differentiation factor 1 (Gdf1), another member of the TGF-beta family, left-sided Nodal is transmitted to the lateral plate mesoderm (LPM), an embryonic structure anterior to the node (Rankin et al., 2000). The Nodal signaling pathway (see Figure 4) is only activated in the left LPM, expressing *Nodal*, as well as *Lefty2*, and *Pitx2*. *Lefty2* encodes a protein that suppresses the Nodal pathway in the right LPM and thereby maintains asymmetry (Meno et al., 2001; Sakuma et al., 2002). Finally, *Pitx2* encodes a transcription factor that remains asymmetrically expressed during the development of the heart and other organs and plays a direct role in their asymmetric morphology (Piedra et al., 1998). Research in zebrafish suggests that prior to cilia movement, Atp6ap1b, a protein involved in ATPase proton pumps, regulates the establishment of the ciliated organ (Kupffer’s vesicle in zebrafish) (Gokey et al., 2015), while the Wnt (wingless-related integration)/beta-catenin pathway regulates cilia length and number (Zhu et al., 2015) and functioning of the Nodal signaling pathway (Hüsken and Carl, 2013). Evidence that the Nodal signaling pathway also corresponds to visceral asymmetry development in humans is given by a strong overlap of genes involved in the Nodal signaling cascade and genetic variants involved in disorders characterized by abnormal asymmetry of human internal organs. In line with findings in mice and chicks, among the genes identified to be involved in laterality defects in humans are *NODAL*, *LEFTY2*, and *GDF1* (Shiraishi and Ichikawa, 2012; Deng et al., 2015).

Besides its involvement in visceral asymmetry development, the Nodal signaling cascade affects structural hemispheric asymmetries in zebrafish. The zebrafish epithalamus consists of bilateral habenular nuclei and the pineal complex containing the medial epiphysis and the left-hemispheric parapineal nucleus (Roussigne et al., 2012). About 20 h after fertilization, genes involved in the Nodal pathway, such as *lefty1* and *pitx2c*, are expressed in the structure later developing into the left epithalamus. Experimentally manipulated symmetrical expression of Nodal genes as well as the absence of Nodal gene expression does not prevent parapineal migration. However, left- and right-sided parapineal nuclei are equally distributed, and the habenular nucleus is larger on the side containing the parapineal nucleus (Concha et al., 2000). Thus, Nodal signaling is responsible for the determination of epithalamic asymmetry direction rather than for establishing asymmetry *per se* (Roussigne et al., 2012). In wild-type zebrafish, the initial symmetry break is induced by the fibroblast growth factor (FGF) pathway, which causes parapineal precursor cells to migrate toward the left hemisphere. Interestingly, the *SETDB2* gene, a candidate gene for handedness, encodes for a methyltransferase that suppresses *fgf8* expression (Ocklenburg et al., 2016a). In zebrafish, no epithalamic asymmetry develops when Fgf8 function is inactivated (Regan et al., 2009), indicating an overlap of molecular mechanisms involved in the ontogenesis of hemispheric asymmetries in zebrafish and humans in the FGF pathway.

Another line of evidence suggests an involvement of the Nodal pathway in human handedness ontogenesis. *PCSK6*, mentioned above as one of the key candidate genes for handedness (Scerri et al., 2011; Arning et al., 2013), has been shown to play a role in left-right determination and the Nodal pathway in mice. At the structural level, a loss-of-function mutation of *pcsk6* leads to defects in the development of the left-right axis such as right-sided stomach, spleen, or pancreas. Moreover, *nodal, pitx2*, and *lefty* are expressed in both the left and the right LPM, suggesting that their usual asymmetric expression patterns are regulated by Pcsk6 (Constam and Robertson, 2000). In humans, *PCSK6* is most strongly expressed in the liver and spinal cord as well as the *corpus callosum*, the largest commissure connecting the hemispheres (Johnson et al., 2003). Based on the findings of *PCSK6* being involved in visceral asymmetry development, other genes causing asymmetry defects when knocked out in mice have been examined with respect to hand performance (Brandler et al., 2013). The most common task used for the determination of hand performance is the Pegboard task. Participants are instructed to move pegs from one row of holes to another with either the left or the right hand. A quantitative measure of fine motor skill is obtained by relating the times required to complete left- and right-hand trials resulting in the so-called PegQ measure (Ocklenburg et al., 2014a). While *PCSK6* again showed the strongest association with PegQ in a cohort selected for reading disability, *PKD2*, meiosis-specific structural protein (*MNS1*), regulatory factor X 3 (*RFX3*), and GLI family zinc finger 3 (*GLI3*) were also among the top hits. The strongest association in a general population cohort was found for a SNP upstream of the Glypican 3 gene (*GPC3*), whose disruption in mice causes lung and heart asymmetry defects. Moreover, genes involved in double outlet right ventricle, heterotaxia, and *situs inversus* (mirror reversal of viscera) were significantly overrepresented in genes associated with hand skill in both cohorts (Brandler et al., 2013). Overall, there is evidence that the molecular pathways controlling visceral laterality may be partly contributing to handedness. As genes involved in ciliopathies are associated with the early development of brain midline structures such as the *corpus callosum* and the cerebellar vermis, molecular mechanisms determining visceral asymmetries might be reused for brain midline structures and therefore affect behavioral laterality (Brandler and Paracchini, 2014).

A recent study investigated structural and functional asymmetry in *situs inversus* subjects with and without primary ciliary dyskinesia (PCD), a recessive disorder resulting in disruption of motile cilia. The authors found an elevated rate of left-handedness in *situs inversus* subjects without PCD, but not in those with PCD. Moreover, the typical counter-clockwise bending of the brain (Yakovlevian torque) was reversed in *situs inversus* subjects, while structural gray and white matter asymmetries and functional language lateralization were not (Vingerhoets et al., 2018). Subsequent whole genome sequencing of the same sample found a probable monogenetic cause for *situs inversus* in those subjects with PCD, while no candidate mutations were identified for most of the *situs inversus* subjects without PCD (Postema et al., 2018). These findings are in line with a link between handedness and visceral asymmetry that is, however, independent of genes involved in cilia function, indicating different mechanisms for different asymmetry phenotypes (Vingerhoets et al., 2018).

### Hemispheric Asymmetries in Gene Expression

The comprehensive literature on visceral asymmetry development in mice and chicken as well as on the ontogenesis of hemispheric asymmetries in zebrafish suggests that besides genetic variants associated with hemispheric asymmetries in humans, gene expression patterns might also play a decisive role. Thus, the reports of structural hemispheric as well as behavioral asymmetries already being apparent in the human fetus set the starting point for the examination of lateralized gene expression in the human fetal brain. Sun et al. (2005) found consistent asymmetrical gene expression between the left and right fetal perisylvian cortex. One of the consistently asymmetrically expressed genes was Lim Domain Only 4 (*LMO4*). Knockdown of *Lmo4* expression in the right anterior cortex in mice resulted in reduced right-hemispheric neuron number, thinner right- than left-hemispheric axonal projections, and a rightward shift of paw preference (Li et al., 2013). As, in humans, the corticospinal tract connecting precentral gyrus and spinal cord does not reach the spinal cord at this early fetal developmental stage (ten Donkelaar et al., 2004), gene expression asymmetries in the developing motor cortex are unlikely the underlying factor for early indications of motor asymmetries. Ocklenburg et al. (2017) found pronounced gene expression asymmetries in the fetal spinal cord. In line with the finding that most fetuses show more right- than left-sided arm movements, the majority of asymmetrically expressed transcripts was expressed more strongly in the right spinal cord. These findings suggest that asymmetrical gene expression in the spinal cord induces asymmetries in motor output that lead to use-dependent plasticity processes in the brain (Ocklenburg et al., 2017).

A major limitation of studies on gene expression in human tissue is the fact that the impact of gene expression on behavioral outcomes is impossible to determine. However, lateralized gene expression has successfully been associated with lateralized behavior in animal models. Grabrucker et al. (2017) reported asymmetrical expression of the candidate genes found by Sun et al. (2005) in the left and right hemispheres of wild-type mice. In the T-maze test, these animals displayed a strong rightward bias. In contrast, prenatal zinc-deficient mice, a mouse model of autism spectrum disorder (ASD), displayed an absence of lateralized gene expression as well as an absence of side preference in the T-maze test (Grabrucker et al., 2017). More direct evidence for an impact of gene expression asymmetries on behavior comes from a study investigating predation behavior in cichlid fish (Lee et al., 2017). While some individuals preferentially attack the left side of their prey (associated with a right-turn, therefore called right-handed), others show the opposite left-handed pattern and only few are non-lateralized. The authors determined individual laterality indices based on the number of left- and right-sided attacks. Gene expression was determined for the left and right tectum opticum, telencephalon, and hypothalamus. Although, in each of the three brain structures, more than 300 genes showed nominally significant left-right differences in their expression patterns, none survived correction for multiple testing. For each gene and brain structure, the fold change of expression between the left and right hemisphere was linked to the behavioral laterality index. In the tectum opticum, 140 genes showed a linear relationship between gene expression asymmetry and behavioral asymmetry during predation. Most of these genes were upregulated in the hemisphere facing toward (ipsilateral to) the prey. In contrast, the 173 genes showing a linear relationship between gene expression asymmetry in the telencephalon and behavioral asymmetry were mostly upregulated in the hemisphere not facing (contralateral to) the prey. Interestingly, one of the genes displaying this pattern was *lrrtm1*, a candidate gene for handedness (Francks et al., 2007). In the hypothalamus, the 79 genes showing a linear relationship between gene expression asymmetry and behavioral asymmetry were also mostly upregulated in the hemisphere contralateral to the prey (Lee et al., 2017). Although this study does not allow for causal conclusions, it is a hint toward an impact of lateralized gene expression on lateralized behavior. Importantly, this relationship was found, although no gene displayed gene expression asymmetry strong enough to survive correction for multiple testing.

### Gene Ontology: Considering Gene Functions

Similar to the findings by Lee et al. (2017) and in contrast to the studies in human CNS tissue (Sun et al., 2005; Ocklenburg et al., 2017), there are a number of studies not replicating expression asymmetries at the level of individual genes in the human fetal (Johnson et al., 2009; Lambert et al., 2011) or adult brain (Hawrylycz et al., 2012; Pletikos et al., 2014; Muntané et al., 2017). These results might imply that asymmetries in gene expression are strongly development-dependent and can only be detected during critical time frames. Another, yet not contradicting, possibility is that gene expression asymmetries are mostly too subtle to detect at individual gene level, especially when correcting for multiple comparisons. Thus, Karlebach and Francks (2015) performed a reanalysis at the level of functional gene groups instead of individual genes, assuming that subtle expression asymmetries of individual genes might translate to strong expression asymmetries at the level of functional gene groups. Lateralized gene expression was found for several functional gene groups involved in signal transmission in the nervous system (Karlebach and Francks, 2015). In a comparative study, Muntané et al. (2017) investigated gene expression in the left- and right-hemispheric ventrolateral prefrontal cortex, superior temporal cortex, and primary motor cortex in humans and macaques. No gene was asymmetrically expressed between the hemispheres after correction for multiple testing. However, weighted gene co-expression network analysis, which clusters genes into modules based on correlations of expression patterns, revealed modules that showed different expression levels between hemispheres in all three cortical areas in humans. These asymmetric modules contained several candidate genes involved in brain asymmetry (e.g., *AR*, *LEFTY1, LMO4,* and *PCSK6*). Moreover, these modules were enriched for functional gene groups such as receptor activity in the superior temporal cortex and locomotion in the primary motor cortex. Interestingly, no module showed differential expression levels between the left and right macaque cortex (Muntané et al., 2017). Thus, the lack of expression asymmetries at the level of individual genes does not necessarily indicate the absence of gene expression asymmetries at the level of functional gene groups.

To determine whether early structural and behavioral asymmetries in human prenatal development are preceded by early asymmetrical gene expression in the CNS, de Kovel et al. (2017) compared gene expression in the left and right human fetal spinal cord and hindbrain at 6–10 gestational weeks. In contrast to the results reported by Ocklenburg et al. (2017), no gene showed individual expression asymmetry after controlling for multiple comparisons. However, functional analysis revealed leftward lateralization of gene groups involved in glutamate receptor signaling and neurotransmitter transport in the human fetal spinal cord. As the expression of both gene groups increases during fetal development, the leftward lateralization was interpreted as the left spinal cord maturing faster than the right. In contrast, the functional gene groups ‘mRNA metabolism’, ‘DNA strand elongation’, ‘chromosome segregation’, and ‘protein translation’ showed rightward lateralization. The expression of these functional gene groups decreases in the course of fetal development, which is also consistent with the assumption of the left spinal cord outpacing the right spinal cord. This pattern was found to be reversed in the hindbrain, consistent with the cross-over of nerve tracts in the inferior hindbrain (de Kovel et al., 2017). In a subsequent study, de Kovel et al. (2018) complemented this dataset with left and right midbrains and forebrains at 7 gestational weeks. At this stage of development, the midbrain showed a similar pattern to the hindbrain with an advanced maturation rate in the right hemisphere. In contrast, the forebrain showed no differences in maturation rates; however, genes expressed in the left forebrain were enriched in the functional gene group ‘cerebral cortex neuron differentiation’, while ‘extracellular structure organization’ was enriched in the right forebrain. The authors performed the same analyses on brain samples obtained at 9–15 gestational weeks with finer subdivisions. Similar to hindbrain and midbrain at earlier developmental stages, they confirmed faster right-hemispheric maturation for the right cerebral cortex (excluding the temporal lobe). In contrast, the left diencephalon, temporal lobe, basal ganglia, and choroid plexus of the lateral ventricle showed faster maturation than their right-hemispheric counterparts. This finding is especially interesting for the choroid plexus as the left-faster-than-right maturation rates at 9–16 gestational weeks are in line with the left-larger-than-right structural asymmetry at 11–13 gestational weeks (Abu-Rustum et al., 2013). This structural asymmetry has been suggested to underlie structural asymmetries in the temporal lobe (Corballis, 2013), a brain region that also shows left-faster-than-right maturation rates (de Kovel et al., 2018).

## The Role of Epigenetic Regulation

The described gene expression asymmetries in early human CNS development raise the question of the underlying molecular factors. Gene expression includes the transcription and translation of a gene, affecting the encoded protein products. Epigenetic mechanisms summarize several chemical modifications to the DNA or proteins involved in DNA packaging that regulate the accessibility of so-called transcription factors to the DNA, which results in activation or repression of transcription (Zhang and Meaney, 2010). Only about 1.2% of the human genome is translated into proteins (Mattick and Makunin, 2006), whereas non-coding DNA has historically been considered as “junk DNA.” However, starting with the Nobel Prize-awarded work of Jacob and Monod (1961), research has shown that an important function of non-coding DNA, such as promoter regions of genes, is the regulation of transcription and translation. The most investigated and best understood form of epigenetic mechanisms is DNA methylation, the addition of a methyl group directly to the DNA, more specifically to the 5-position of cytosine guanine (CpG) dinucleotides. Within promoter regions, stronger DNA methylation typically represses transcription of that gene (Jaenisch and Bird, 2003). The complex interplay of epigenetic mechanisms has been shown to play a key role in pre- and postnatal brain development (Bale, 2015). While an important function of epigenetic regulation is tissue-specific differentiation of cells and therefore a mechanism by which cells with an identical genotype are able to take different forms, epigenetic mechanisms can also change as a function of environmental factors (Tammen et al., 2013).

### Epigenetics in the Development of Hemispheric Asymmetries

A number of environmental factors have more or less consistently been shown to affect handedness (de Kovel et al., 2019). We previously reviewed the empirical evidence and suggested that the epigenetic regulation might be an underlying factor connecting environment and phenotype (Schmitz et al., 2017b). In the human spinal cord, Ocklenburg et al. (2017) could show that approximately one third of variance in asymmetrical gene expression could be explained by epigenetic regulation. Interestingly, these epigenetic factors were enriched in the TGF-beta signaling pathway, adding additional evidence for an overlap between molecular mechanisms involved in the development of visceral asymmetry and hemispheric asymmetries. This is also in line with the association of *SETDB2* with handedness in adults, as the protein encoded by this gene not only regulates left-right asymmetry in zebrafish but also regulates gene expression epigenetically (Ocklenburg et al., 2016a). As access to DNA in healthy humans is limited to peripheral tissues, the influence of epigenetic modulation on hemispheric asymmetries in adults has so far only been investigated in buccal samples. Although DNA methylation is tissue-specific, it has been proposed that its degree in peripheral tissue can be interpreted as a biomarker for CNS-related phenotypes (Klengel et al., 2014; Freytag et al., 2017). Leach et al. (2014) investigated DNA methylation in 19 CpG sites in the *LRRTM1* promoter region. Principal component analysis revealed a block of CpG sites that was negatively correlated with strength of hand preference in the overall sample as well as in female participants. Thus, stronger DNA methylation in these CpG sites was associated with a tendency toward ambidexterity. The authors conclude that this finding suggests an effect of environmental factors on handedness *via* epigenetic modulation (Leach et al., 2014). We recently found that DNA methylation in promoter regions of genes asymmetrically expressed in the human fetal CNS predicts handedness direction (Schmitz et al., 2018a). Moreover, the amount of experienced birth stress in pre- and perinatal development was correlated with DNA methylation in *NEUROD6*, whose expression is tripled in the left compared to the right perisylvian cortex at 12 gestational weeks (Sun et al., 2005). Moreover, we found an association between language lateralization in the dichotic listening task and DNA methylation in the *KIAA0319* promoter region in buccal cells. The effect was not found for language lateralization *per se*, but for the conditions reflecting attentional modulation, indicating *KIAA0319* as an epigenetic marker for cognitive control processes. As *KIAA0319* is a major candidate gene for dyslexia and presumably involved in the ontogenesis of visceral asymmetries, this finding is partly in line with an overlap of genes involved in visceral asymmetries and the ontogenesis of hemispheric asymmetries (Schmitz et al., 2018b).

### Transgenerational Epigenetic Inheritance: Is That Possible?

We recently argued that heritable epigenetic mechanisms might partly account for the missing heritability of handedness (Schmitz et al., 2017b). However, since the existence of transgenerational epigenetic inheritance in humans is highly controversial (Babenko et al., 2015), how can epigenetic mechanisms contribute to lateralization at the population level?

#### Germ Line Epigenetic Inheritance

**Figure 5:**
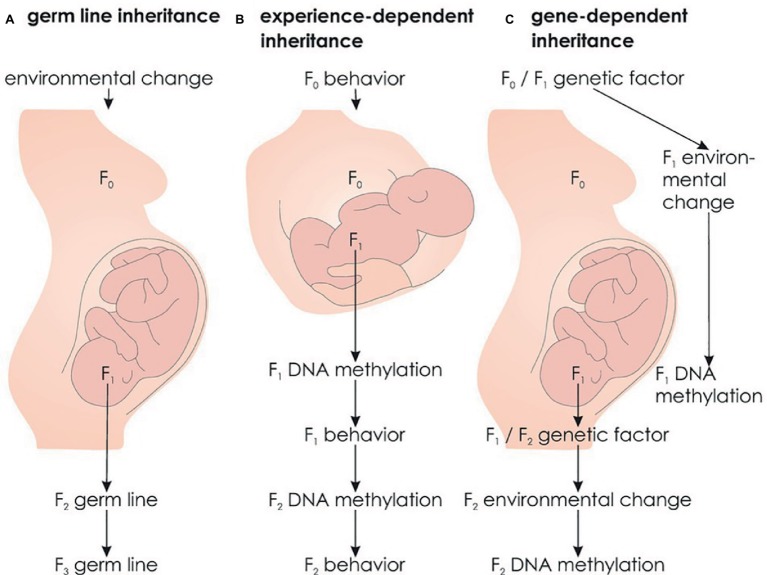
Alternative forms of transgenerational epigenetic inheritance. **(A)** Germ line epigenetic inheritance: An environmental factor acts on the F_0_ generation and induces an epigenetic state that is transmitted to subsequent, unaffected individuals *via* the germ line. **(B)** Experience-dependent epigenetic inheritance: Maternal behavior induces an epigenetic state in the offspring (F_1_) that in turn influences F_1_ behavior toward its offspring (F_2_) transmitting behavior and epigenetic states across generations. **(C)** Gene-dependent epigenetic inheritance: A genetic factor modulates the probability of an environmental factor that influences F_1_ epigenetic states. As F_1_ likely transmits the genetic factor to F_2_, epigenetic states are transmitted across generations.

Transgenerational epigenetic inheritance typically refers to direct transmission *via* the germ line, meaning that an environmental factor influences the epigenetic state in the F_0_ generation, which is transmitted to subsequent generations *via* the germ line (Danchin et al., 2011). There are two criteria to the demonstration of germ line epigenetic inheritance. First, the environmental factor has only affected the F_0_ generation, but not subsequent generations. Second, because germ cells might be directly influenced by the environmental factor, germ line epigenetic inheritance requires transmission over at least three generations in the female or at least two generations in the male germ line (see Figure 5A; Crews, 2008).

**Figure 6:**
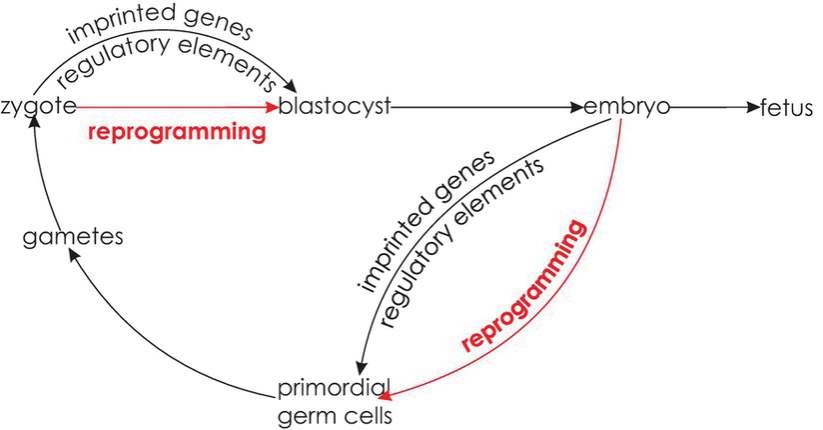
Epigenetic reprogramming. Reprogramming occurs shortly after fertilization in the zygote and in the primordial germ cells of the developing embryo. At both stages, some epigenetic states are able to escape reprogramming.

However, due to the phenomenon of reprogramming, it has long been assumed that germ line epigenetic inheritance is not possible. Reprogramming refers to the erasure of epigenetic signatures between generations at two times during development. First, reprogramming takes place shortly after fertilization in the zygote. Second, epigenetic signatures are removed in primordial germ cells of the developing embryo that later develop into gametes. These reprogramming events ensure restoring of pluripotency of germ cells and zygotes (Babenko et al., 2015). The fact that epigenetic states are erased during embryonic development conflicts with the idea that epigenetic states can be inherited. However, it has been shown that some epigenetic states escape both reprogramming processes, such as imprinted genes (Bartolomei, 2009) and regulatory elements (see Figure 6; Hackett et al., 2013), potentially leading to transgenerational epigenetic inheritance over the germ line.

In mammals, transgenerational epigenetic inheritance has mainly been reported in mice and rats, while it remains controversial in humans (Ambeskovic et al., 2017a) due to the correlational nature of studies reporting these effects. A group of epidemiological studies suggest that *in utero* exposure to the Dutch famine in 1944 and 1945 is associated with less DNA methylation of the insulin-like growth factor 2 (*IGF2*) gene (Heijmans et al., 2008) and affects birthweight and height of grandchildren (Painter et al., 2008; Veenendaal et al., 2013). However, no data are currently available for the critical F_3_ generation. Another group of studies examined a cohort from Överkalix, a small isolated village in Sweden with detailed records on food availability (Bygren et al., 2001). A surfeit of food during paternal grandfathers’ childhood was associated with a fourfold increase of diabetes in grandchildren (Kaati et al., 2002, 2007). As this effect was transmitted through grandfathers and fathers, these studies indicate sperm-mediated transgenerational epigenetic transmission over the germ line (Pembrey, 2002). Based on these epidemiological studies and research in rodents, transgenerational epigenetic effects on brain functions transmitted over the germ line (see Figure 5A) have been postulated to exist in humans as well (Bohacek et al., 2013). However, there is currently no indication of a unique environmental factor in preceding generations to affect an individual’s handedness.

#### Experience-Dependent Epigenetic Inheritance

**Figure 7:**
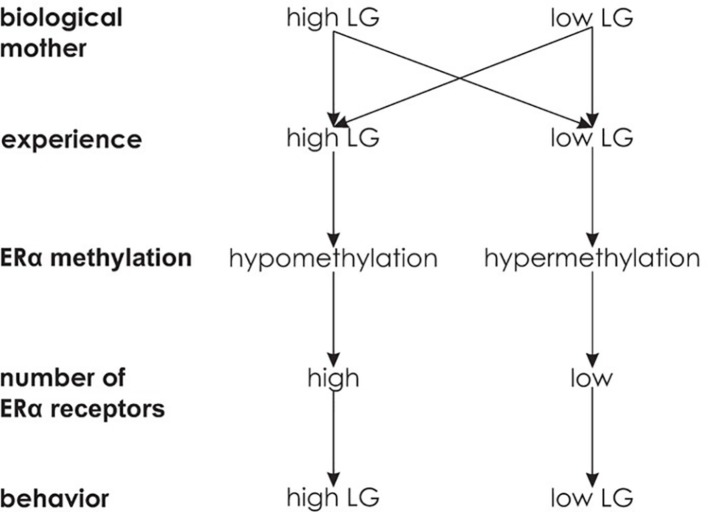
Experience-dependent epigenetic inheritance. Independent of licking and grooming behavior (LG) of the biological mother, the experience of high LG is associated with a large number of ERα receptors and high LG behavior, while the experience of low LG behavior is associated with a low number of ERα receptors and low LG behavior.

There are alternative forms of transgenerational epigenetic inheritance. In female rats, the experience of high maternal licking and grooming (LG) behavior leads to relative hypomethylation of the estrogen receptor alpha (ERα) promoter region in the hypothalamus, resulting in more pronounced production of ERα receptors. In contrast, low LG experience leads to relative ERα promoter hypermethylation and less ERα receptors. The increase or decrease in ERα receptors causes the offspring (F_1_) to show more or less LG behavior toward their own offspring (F_2_), respectively (see Figure 7).

Importantly, cross fostering confirmed the experience of LG behavior being causative for ERα expression. Biological offspring of high LG mothers that was cross fostered to low LG mothers had a similarly small amount of ERα receptors in the hypothalamus as regular offspring of low LG mothers and thus showed low LG behavior themselves. In contrast, biological offspring of low LG mothers cross fostered to high LG mothers showed a high amount of ERα receptors in the hypothalamus and high LG behavior as adults (Champagne et al., 2006). Overall, this indicates that an epigenetic state influences an organism’s behavior such that it induces the same epigenetic state and behavior in its offspring. Thus, in contrast to germ line epigenetic transmission, experience-dependent epigenetic transmission induces recreation of epigenetic states in every new generation (see Figure 5B). While parental behavior and epigenetics interactively influence inheritance, changes in the environment can interrupt the transmission across generations (Danchin et al., 2011).

Interestingly, paw preference and hemispheric dominance in terms of dendritic complexity and spine density have been compared in F_4_ generations of rats with ancestors being exposed to stress multigenerationally (female rats in the F_0_–F_3_ generation had been exposed to stress) or transgenerationally (only female rats in the F_0_ generation had been stressed). While both transgenerational and multigenerational stress led to an increase in left paw preference in the F_4_ male offspring, this shift was more pronounced and significantly different from non-stressed controls in the multigenerationally stressed individuals. Moreover, a preference for the left paw was associated with increased dendritic complexity and spine density in the right parietal cortex (Ambeskovic et al., 2017b). As stress during pregnancy has been shown to affect maternal behavior (Yao et al., 2014), maternal behavior might have induced experience-dependent epigenetic inheritance of hemispheric asymmetries.

Experience-dependent epigenetic inheritance is also conceivable in humans. For example, an individual’s attachment to their mother is predictive of their infant’s attachment to them, and the experience of less parental care is reflected in lower sensitivity toward the own children (Champagne, 2008). Applied to handedness, parental (F_0_) behavior might lead to specific DNA methylation patterns in the offspring (F_1_), initiating the same behavior toward their children (F_2_). This in term might result in transmission of DNA methylation patterns to the F_2_ generation (see Figure 5B).

#### Gene-Dependent Epigenetic Inheritance

The findings of environmental factors involved in handedness (Schmitz et al., 2017b, 2018a) open up another possibility of transgenerational epigenetic inheritance. Coren (1995) suggested that the familial transmission of left-handedness might indicate a genetic origin but could also be explained by the possibility that left-handed mothers have experienced more birth complications than right-handed mothers. The author suggests that the experience of a complicated birth might lead to non-specified “internal conditions,” which elevate the probability of complications during delivery and left-handed offspring. This hypothetical scenario was partly confirmed as left-handed mothers reported more birth complications (especially breech birth and prolonged labor) and more left-handed offspring than right-handed mothers, while left-handed fathers did not play a role in the number of birth complications or left-handed offspring. However, the author assumed that left-handed mothers had experienced more complications during their own birth without empirically testing this assumption (Coren, 1995). It might be possible that there is interindividual variability in proneness to the risk of a certain environmental event (e.g., birth complications) due to the genetic setup. The environmental factor might be associated with epigenetic modifications (for example, in the *NEUROD6* promoter region, Schmitz et al., 2018a) eventually leading to changes in behavior (handedness). This third possibility of transgenerational epigenetic inheritance (see Figure 5C) requires three criteria.

1. There is a genetic risk for an environmental factor
  Individuals who were delivered in breech position are more likely to deliver in breech position themselves in their first pregnancy than individuals who were delivered in cephalic position (odds ratio = 2.3) (Nordtveit et al., 2008). Moreover, the odds ratio of breech delivery after one previous breech delivery is elevated to 4.3 (Albrechtsen et al., 1998). Interestingly, the usual rightward head turning preference of fetuses in a cephalic position is not present in fetuses in breech position that rather show a head midline preference (Fong et al., 2005). As direction of head turning is strongly associated with later handedness (Coryell and Michel, 1978; Michel, 1981; Konishi et al., 1986; Ocklenburg et al., 2010), a genetic influence on fetal position might be associated with ontogenesis of hemispheric asymmetries. Mothers who were born prematurely have an enlarged risk of preterm delivery compared to women born at term (Porter et al., 1997) with an odds ratio of 1.5 (Wilcox et al., 2008). Preterm birth seems to be a heritable trait with a polygenic cause (Chaudhari et al., 2008) with twin studies estimating genetic factors to account for 30% of the phenotypic variance in gestational age at delivery (Kistka et al., 2008). Similar results have been reported for birthweight (Clausson et al., 2000) where genetic factors of the fetus accounted for 31% of phenotypic variance and maternal genetic factors accounted for 22% of the variation in birth weight (Lunde et al., 2007). Overall, pronounced genetic factors seem to influence different types of birth complications.
2. This environmental factor affects DNA methylation
  Prenatal stress has been shown to exert long-term effects on the hypothalamic-pituitary-adrenocortical (HPA) axis. For example, the experience of severe stress *in utero* leads to a stronger cortisol response to the Trier Social Stress Test (TSST) in young adults as compared to control subjects without prenatal stress experience (Entringer et al., 2009). Similar effects have been reported for prenatal stress and cortisol reactivity toward temporary stressors such as vaccination or maternal separation (Tollenaar et al., 2011). Several studies have also reported an effect of prenatal stress on basal cortisol levels (Glover et al., 2010). Recent research suggests that prenatal stress induces its associated outcomes *via* epigenetic modification (Glover, 2015). For example, DNA methylation in genes associated with HPA axis regulation is altered in cord blood and placenta in individuals with chronic stress experience during pregnancy compared to controls (Kertes et al., 2016). The association between prenatal stress and DNA methylation in the glucocorticoid receptor (GR) gene (*NR3C1*) (Perroud et al., 2014) has been confirmed by meta-analysis (Palma-Gudiel et al., 2015). Birth stress might also modify fetal DNA methylation *via* its associated enhanced levels of oxidative stress (Saphier et al., 2013; Ávila et al., 2015). Interestingly, cortisol and other glucocorticoids released by acute or chronic stress might also play a role in modulating hemispheric asymmetries, which often results in stronger right-hemispheric involvement (Ocklenburg et al., 2016b).
3. DNA methylation affects the phenotype
  As mentioned above, the study by Leach et al. (2014), as well as our own work (Schmitz et al., 2018a,b), provides first hints that epigenetic markers for handedness and language lateralization can be found in buccal cells. Moreover, findings from spinal cord tissue in the human fetus suggest an involvement of epigenetic mechanisms in asymmetrical gene expression, potentially leading to motor asymmetries (Ocklenburg et al., 2017).

Overall, gene-dependent epigenetic inheritance (see Figure 5C) is an alternative mechanism for epigenetic transmission that is worth being empirically tested. Although this proposed mechanism is based on an initial genetic factor, it would not necessarily be captured by classic molecular genetic studies. For example, genetic polymorphisms involved in certain birth complications would not be captured by GWAS on the handedness phenotype, as they might only exert their influence on handedness depending on the interplay with environmental factors. This is also illustrated by the special case of twins. Sharing the same genetic background, MZ twins have the same genetic risk of birth complications. However, this does not necessarily mean that both co-twins are equally affected by birth complications. For example, 75% of MZ twins are monochorionic, i.e., share a placenta (Cordero et al., 2005). Unequal placenta sharing, i.e., one co-twin receives blood from more than 60% of the placenta, has repeatedly been shown to affect discordance of birth weight in MZ twins (Fick et al., 2006; Zhang et al., 2013). As the placenta is crucial for providing the embryo or fetus with nutrients (Nugent and Bale, 2015), these findings argue for different intrauterine environments even in MZ twin pairs. These environmental differences might underlie the reported differences in DNA methylation patterns in DZ and MZ twin pairs immediately after birth (Ollikainen et al., 2010; Gordon et al., 2012). Interestingly, preeclampsia, one of the most fatal birth complications, is associated with reduced levels of DNA methylation and increased expression of genes involved in the TGF-beta signaling pathway such as *PITX2* (Martin et al., 2015).

Moreover, both MZ and DZ twins are more likely to experience a complicated birth and to display lower weight and shorter gestational age at birth. This is in line with a Finnish large-scale study reporting that the often described increased probability of left-handedness in twins is absent after controlling for these two factors, showing that birth weight and gestational age might rather contribute to handedness development than twinning *per se* (Heikkilä et al., 2015). As the probability to conceive twins is partly genetically determined (Machin, 2009; Painter et al., 2010; Mbarek et al., 2016), a genetic factor might modulate the probability of certain prenatal environmental factors such as reduced placental blood supply and thus access to important nutrients (Nugent and Bale, 2015) or perinatal complications in general. As mentioned above, differences in prenatal environment between MZ twins likely induce the observed differences in DNA methylation at birth (Gordon et al., 2012), which might have effects on the ontogenesis of hemispheric asymmetries. Thus, an identical genetic background does not contradict the idea of gene-dependent epigenetic inheritance, as genetic factors do not determine prenatal environment, but modulate it in a probabilistic way.

Overall, a complex interplay of genetic, environmental, and epigenetic factors might contribute to handedness development. A similar mechanism has been shown to lead to the ontogenesis of visual processing asymmetries in birds. In pigeons, the left hemisphere outperforms the right hemisphere in categorization and discrimination tasks. This functional hemispheric asymmetry can be prevented by incubating pigeons in darkness thereby withdrawing the environmental factor light. This is possible because birds occupy an asymmetrical position within the egg. While the right eye faces toward the eggshell, the left eye is covered by the own body. Thus, a genetically determined body position results in an environmental factor (light exposure) stimulating the right, but not the left eye, through the semi-translucent eggshell initiating a cascade of molecular events eventually leading to asymmetries at the behavioral level. While epigenetic mechanisms are thought to play a role in this cascade of molecular events, this possibility has not yet been confirmed by empirical testing (Güntürkün and Ocklenburg, 2017; Ocklenburg and Güntürkün, 2018). In humans, a genetic predisposition might lead to an enhanced probability of birth complications that then induces epigenetic modification of the *NEUROD6* promoter region or other genes. As mentioned above, this mechanism is most likely to affect DNA methylation in brain cells, while our results in buccal cells are better described as an epigenetic signature for handedness in non-neuronal tissue.

The potential mechanism of gene-dependent epigenetic inheritance for familial transmission of handedness raises the question if a similar mechanism is conceivable for language lateralization. However, environmental factors involved in the development of language lateralization have been investigated sparsely, and we found an effect of DNA methylation on cognitive modulation of language lateralization, but there was no effect on language lateralization *per se* (Schmitz et al., 2018b). Thus, further research is needed in order to come up with a mechanistic model on the ontogenesis of language lateralization.

## Perspectives

Future studies should investigate different forms of hemispheric asymmetries and their relations with epigenetic modifications and environmental factors such as birth complications – not only in the individual but also transgenerationally. While epigenetic research in humans remains correlative, animal models allow for experimental manipulation and thus for uncovering causal mechanisms. Knowledge on molecular mechanisms involved in hemispheric asymmetries can benefit from experimental manipulation of prenatal conditions in rats and mice. These animal models also allow for the analysis of behavior, gene expression, and DNA methylation in the left and right hemispheres. Moreover, transgenerational effects can be investigated in more detail in animal models (Ambeskovic et al., 2017b).

In humans, large-scale GWAS are needed to uncover the genetic background of hemispheric asymmetries and their association with visceral asymmetries. Two GWAS on hand preference have recently been performed in the UK Biobank cohort. Significant associations were shown for *MAP2* (de Kovel and Francks, 2019; Wiberg et al., 2019) as well as *MAPT* and *TUBB* (Wiberg et al., 2019). Interestingly, rs199512 showed genome-wide significance for hand preference and was strongly associated with structural connectivity in the superior longitudinal fasciculus (Wiberg et al., 2019). However, neither an association with known candidate genes nor an overrepresentation of genes involved in visceral asymmetry could be replicated. These findings are in line with different genetic factors involved in handedness if measured continuously or categorically (de Kovel and Francks, 2019). Large samples phenotyped with both hand preference and hand performance are needed to reach conclusive results.

Future research should also investigate whether an interplay of genetic, environmental, and epigenetic factors contributes to asymmetry at the population level or at the individual level. While individual level asymmetry is characterized by pronounced strength of asymmetry in all individuals, but independent from direction of asymmetry, population level asymmetry is characterized by both strength and direction of asymmetry (Ströckens et al., 2013). For example, variation in *PCSK6* has been associated with strength, but not direction of handedness, suggesting that strength and direction represent different phenotypes affected by distinct molecular pathways (Arning et al., 2013). This is also in line with the Nodal pathway being associated with direction of asymmetry, but not asymmetry *per se*, indicated by individual level, but no population level asymmetry when Nodal gene expression is absent or symmetrical (Concha et al., 2000). In the a*via*n visual system, it has been shown that the direction of asymmetry can be reversed by environmental factors, supporting an interplay of genetic, environmental, and potentially epigenetic factors affecting the direction of asymmetry (Manns, 2006). However, more research is needed to elucidate these mechanisms.

It has to be kept in mind that just as patterns of behavioral lateralization differ between humans and other vertebrates (Ströckens et al., 2013; Ocklenburg et al., 2013d), its underlying molecular mechanisms in animal models might not be identical to humans. However, a well-thought-out combination of experimental manipulation and in-depth analysis of epigenetic mechanisms in animal models and correlative research in humans might be the best strategy to reveal important insights into the ontogenesis of hemispheric asymmetries (Alqadah et al., 2018).

## Author Contributions

JS wrote the manuscript. OG and SO advised on the manuscript and provided critical feedback.

### Conflict of Interest Statement

The authors declare that the research was conducted in the absence of any commercial or financial relationships that could be construed as a potential conflict of interest.
